# Surface Plasmon Enhancement of Eu^3+^ Emission Intensity in LaPO_4_/Ag Nanoparticles

**DOI:** 10.3390/ma13143071

**Published:** 2020-07-10

**Authors:** Sanja Kuzman, Jovana Periša, Vesna Đorđević, Ivana Zeković, Ivana Vukoje, Željka Antić, Miroslav D. Dramićanin

**Affiliations:** VINČA Institute of Nuclear Sciences-National Institute of the Republic of Serbia, University of Belgrade, 11000 Belgrade, Serbia; sanjaculubrk@gmail.com (S.K.); jburojevic@vinca.rs (J.P.); vesipka@vinca.rs (V.Đ.); zekovicivana@gmail.com (I.Z.); ivanav@vinca.rs (I.V.); dramican@vinca.rs (M.D.D.)

**Keywords:** inorganic materials, luminescence, optical properties, plasmonics

## Abstract

A promising way to improve the performance of luminescent materials is to combine them with noble metal nanoparticles. Herein, a set of silver/europium-doped lanthanum orthophosphate (Ag/La_0.95_Eu_0.05_PO_4_) nanostructures with different concentrations of silver nanoparticles were prepared and investigated. The presented overlap between the strongest europium (Eu^3+^) excitation line and the broad silver nanoparticle surface plasmon resonance makes the combination prospective for coupling. X-ray powder diffraction confirmed the monoclinic monazite structure. The transmission electron microscopy revealed particles with a rod-like shape and ~4 aspect ratio. Photoluminescence spectra show characteristic Eu^3+^ ion red emission. One of the requirements for an enhanced luminescence effect is the precise control of the distance between the noble metal nanoparticles and the emitter ion. The distance is indirectly varied throughout the change of Ag nanoparticle concentration in the La_0.95_Eu_0.05_PO_4_ host. The emission intensity increases with the increase in Ag nanoparticles up to 0.6 mol %, after which the luminescence decreases due to the nanoparticles’ close packing and aggregation leading to the displacement of La_0.95_Eu_0.05_PO_4_ from the vicinity of the metal particles and reabsorption of the emitted light. The emission intensity of La_0.95_Eu_0.05_PO_4_ increases more than three times when the Eu^3+^ excitation is supported by the localized surface plasmon resonance in the Ag/La_0.95_Eu_0.05_PO_4_ nanostructures.

## 1. Introduction

Advanced materials are crucial for social and economic development, with applications in industries aimed to meet the challenges of renewable and clean energy, climate changes, national security, as well as human health and welfare. Therefore, the development and exploitation of innovative materials is critical in achieving global competitiveness in the 21st century. To meet, among others, demands for better physical and chemical sensors, brighter phosphors, or biocompatible drug-delivery molecules, material scientists are searching for new molecular combinations and structures [1,2,3,4].

The design and preparation of phosphors, inorganic materials activated by lanthanide (Ln^3+^) or transition metal (TM) ions, with different particle size and/or distinctive morphology is a challenging task that has attracted researchers’ attention. Phosphors are used in a variety of applications, such as displays, lighting, optical sensing (physical and chemical), catalysis, and medicine, just to mention a few [5,6,7]. Phosphor’s luminescent properties depend on the characteristics of the optically active ion and its local environment provided by the host lattice. In recent years, the lanthanum orthophosphate (LaPO_4_), non-toxic, biocompatible, thermally, chemically, and photo-stable material has been recognized as an excellent choice for rare-earth ion doping [8,9,10,11,12]. Due to the similar ionic radii and charge, lanthanum ion (La^3+^) can be easily replaced with different rare-earth (RE^3+^) ions (e.g., Eu^3+^, Dy^3+^, Sm^3+^) at a wide range of concentrations without significantly affecting the lattice structure [13].

The optical properties of luminescent materials can be improved by different approaches, such as by the optimization of the particle size and distribution, morphology, and crystal defects. Moreover, the modification of the local structure based on strategies such as the control of the doping level, cationic, anionic and cationic/anionic substitution, crystal-site engineering, and the mixing of nanophases are also promising routes [14]. To date, to improve and optimize the luminescence properties of LaPO_4_-based phosphors, studies have focused on changing the particle size and shape, designing core-shell structures, surface modification, and co-doping with alkali metals [15,16,17,18,19,20,21,22]. However, Ln^3+^ activated phosphors suffer from the very low absorption in the near-UV and visible spectral regions due to quantum mechanically forbidden f–f electronic transitions. For this reason, they cannot be used for the blue or near-UV excited LEDs unless their luminescence is not appropriately sensitized by co-dopants (for example, Bi^3+^, Ce^3+^), dyes, or plasmon particles. Collective oscillations of surface electrons in noble metal nanoparticles (plasmons) resonantly interact with the incident or emitting radiation, causing an increase in the light absorption cross-section and the radiative rate of an adjacent emitter. Thus, the brightness of Ln^3+^ and TM ion-activated phosphors may be increased through more effective light absorption when they are located in the vicinity of plasmon particles, for example noble metal clusters. Among different plasmonic structures, Ag nanoparticles are particularly useful for the phosphors’ research because their plasmon occurs around 430 nm that is suitable for sensitizing most of the Ln^3+^ activators. To date, there is a number of reports on the phosphor/Ag nanoparticle (NP) systems as a promising combination for the emission enhancement [23,24,25,26,27,28,29,30,31,32]. However, there are only a few reports on the Ag plasmon-enhanced emission of LaPO_4_-based phosphors. Li et al. investigated the enhancement of luminescent properties in inverse opal and silica-coated inverse opal LaPO_4_:Eu^3+^ structures after the addition of Ag nanoparticles [33,34]. For silica-coated inverse opal structures, they reported the enhancement factor of ~7.

The aim of this work was to investigate how co-doping with silver nanoparticles affects the luminescence properties of the La_0.95_Eu_0.05_PO_4_ phosphor. A simple hydrothermal method was used for the preparation of Ag/La_0.95_Eu_0.05_PO_4_ nanostructures with different silver nanoparticles concentrations. Lanthanum orthophosphate and hydrothermal synthesis were chosen due to the low crystallization and reaction temperature which prevents silver nanoparticles from melting and agglomeration [23]. Structural, morphological, and optical properties were reported and discussed. The effect of silver nanoparticles on the luminescence properties of La_0.95_Eu_0.05_PO_4_ is elaborated in terms of Ag NPs optimal concentration and luminescence enhancement factor.

## 2. Materials and Methods

### 2.1. Synthesis of Ag/La_0.95_Eu_0.05_PO_4_ Nanostructures

**Figure 1:**
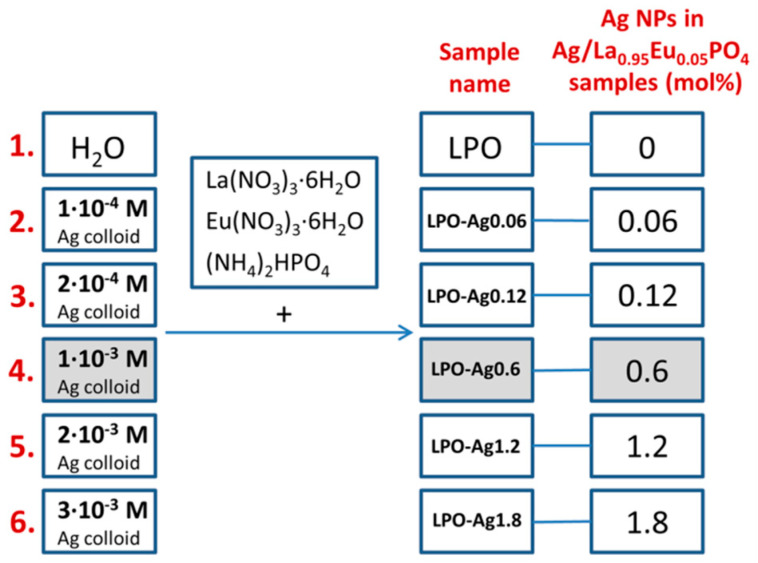
Marking of the La_0.95_Eu_0.05_PO_4_ (LPO) samples with different concentrations of Ag nanoparticles (NPs)**.**

A set of six samples was prepared by the conventional hydrothermal synthesis. Five samples were prepared by using silver colloids of different concentrations (1 × 10^−4^ M; 2 × 10^−4^ M; 1 × 10^−3^ M; 2 × 10^−3^ M; 3 × 10^−3^ M) as a medium, while one sample was prepared in water (see Figure 1 for the sample’s names and Ag NPs mol %). The mole percentages (mol %) of Ag NPs in the samples were calculated relative to the overall content in the Ag/La_0.95_Eu_0.05_PO_4_ nanostructures. According to our previous results [13], there is no concentration quenching in RE^3+^-doped LaPO_4_ NPs and the emission intensity increases with an increase in RE^3+^ dopant concentration up to the full substitution of La^3+^ with RE^3+^. We chose the La_0.95_Eu_0.05_PO_4_ system where the Eu^3+^ emission is strong enough so that the effect of Ag NPs on the luminescence efficiency can be clearly observed.

Synthesis of silver colloid: An appropriate amount of silver nitrate (AgNO_3,_ Merck) was dissolved in the water previously purged with argon for 30 min. The reducing sodium borohydride (NaBH_4_) agent was added to the solution by vigorous stirring. A large excess of NaBH_4_ was required to reduce the silver ions and to stabilize the formed silver nanoparticles. The obtained colloids were left in an argon atmosphere for an additional 30 minutes. The concentrations of obtained silver colloids were: 1 × 10^−4^ M; 2 × 10^−4^ M; 1 × 10^−3^ M; 2 × 10^−3^ M and 3 × 10^−3^ M, and they were used without any further purification.

Synthesis of Ag/La_0.95_Eu_0.05_PO_4_ nanostructures: In the first step, the stoichiometric quantities of lanthanum(III) nitrate hexahydrate (La(NO_3_)_3_·6H_2_O, Alfa Aesar, 99.9%), europium(III) nitrate pentahydrate (Eu(NO_3_)_3_·6H_2_O, Alfa Aesar, 99.9%) and diammonium hydrogen phosphate ((NH_4_)_2_HPO_4_, Alfa Aesar, 98.0%) were dissolved in an appropriate amount of water/silver colloid. The resulting solutions (20 mL) were transferred into a 50 mL Teflon-lined Stainless Steel Autoclave and kept at 140 °C for 30 h, followed by natural cooling to room temperature. Obtained white precipitates were washed with distilled water and ethanol and dried at 40 °C.

### 2.2. Instruments and Measurements

X-ray diffraction (XRD) measurements were performed using the Rigaku SmartLab diffractometer, Rigaku Corporation, Tokyo, Japan. Diffraction data were recorded in a 2θ range from 10° to 90°, counting 0.1°/min in 0.02° steps. Transmission electron microscopy was performed on the FEI TECNAI G2 X-TWIN microscope. Diffuse spectral reflectance measurements were performed on the Thermo Evolution 600 spectrometer equipped with an intergrading sphere, using BaSO_4_ as a blank. The photoluminescent emission spectra were collected using a Fluorolog-3 Model FL3-221 spectrofluorometer system (Horiba-Jobin-Yvon) under continuous excitation using a 450W xenon lamp (λ_ex_ = 393 nm). The photoluminescent excitation spectra were recorded while monitoring emission at λ_em_ = 611 nm.

## 3. Results and Discussion

### 3.1. Structural Analysis

**Figure 2:**
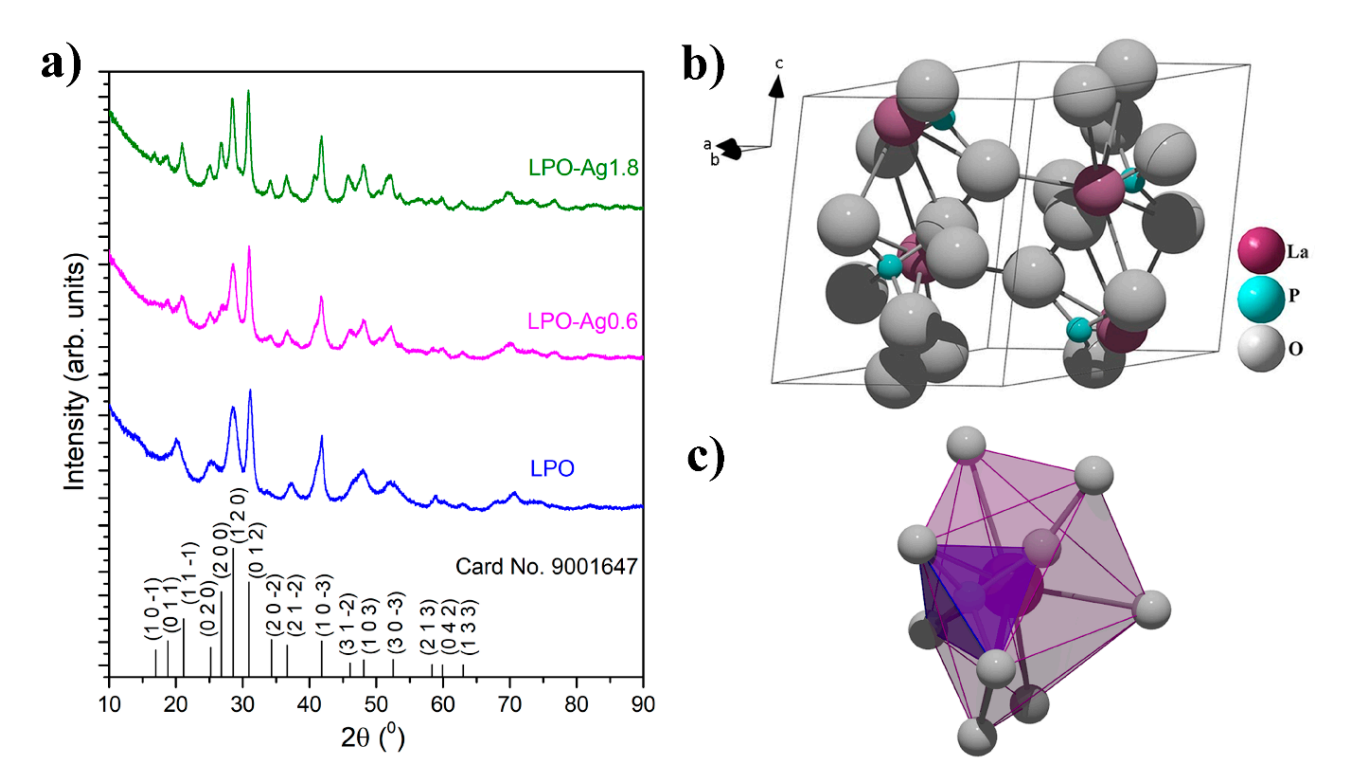
(**a**) XRD patterns of the representative LPO, LPO–Ag0.6, and LPO–Ag1.8 samples. The diffraction peaks are indexed according to the Crystallography Open Database - COD card No. 9001647. Lanthanum phosphate (LaPO_4_); (**b**) unit cell; and (**c**) coordination polyhedron around La^3+^.

X-ray powder diffraction patterns of the representative LPO and two Ag/La_0.95_Eu_0.05_PO_4_ (LPO–Ag0.6 and LPO–Ag1.8) samples confirmed the monoclinic monazite structure with space group P 121/n 1(14) (see Figure 2a, all diffraction peaks are indexed according to the COD card no. 9001647). The absence of impurity phases indicates that the dopant Eu^3+^ ions are successfully incorporated into the LaPO_4_ matrix due to the equal valence (+3) and similar ionic radii between the Eu^3+^ (*a* = 0.112 nm) and La^3+^ ions (*a* = 0.122 nm) [35]. In this crystal structure, the lanthanide ions are coordinated with nine oxygen atoms forming polyhedrons (LaO_9_) that a share corner with PO_4_ tetrahedra in which all four P–O bonds are equivalent (Figure 2b,c) [13,36]. The average crystallite size of ~6 nm was determined using built-in software and was similar for all the samples.

### 3.2. Microstructural Characterization

**Figure 3:**
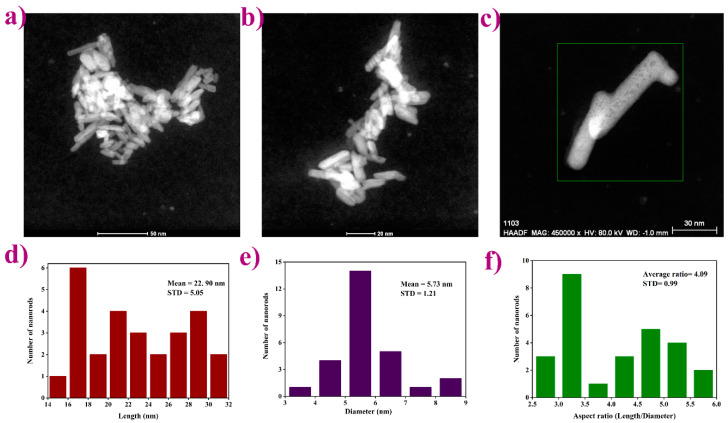
Transmission electron microscopy images showing: (**a**–**c**) anisotropic rod-like shape particles with an aggregation tendency; (**d**–**f**) average length/diameter/aspect ratio histograms.

Transmission electron microscopy images (Figure 3a–c) of the representative LPO–Ag0.6 sample show particles with a rod-like shape, expected for the given monoclinic crystal system. Furthermore, the TEM images reveal an aggregation tendency also found in higher condensed La-based phosphates [37]. An average length/diameter aspect ratio was calculated and found to be around 4 (Figure 3d–f). According to the literature [38,39], when synthesized under alkaline conditions (pH > 7) the particles are sphere-like. On the other hand, when synthesized under acidic conditions (pH < 7), the particles become rod-like with a different length/diameter ratio corresponding to the different pH values. In a strongly acidic solution, rod-like morphology becomes fiber-like having a width of 5–20 nm and a length of several micrometers. Herein, the prepared mildly acidic precursor solutions (pH ~5) yield to, as expected, rod-like particles with ~4 aspect ratio.

### 3.3. Diffuse Reflectance

**Figure 4:**
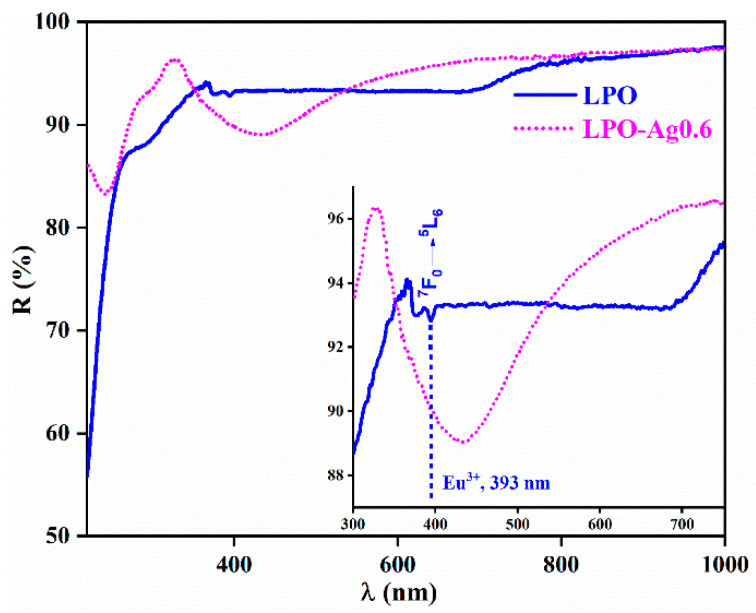
Diffuse reflectance spectra of the representative LPO (blue solid line) and LPO–Ag0.6 (pink-dotted line) sample. Inset: the dip in the reflection spectrum of the LPO–Ag0.6 sample centred at 430 nm is due to Ag plasmon absorption and it overlaps with the ^7^F_0_→^5^L_6_ absorption band of Eu^3+^.

Figure 4 shows the diffuse reflectance spectra of the representative LPO and LPO–Ag0.6 samples. The sample with the Ag NPs clearly shows a typical broad band centred at 430 nm originating from the Ag surface plasmon resonance [40,41]. On the other hand, the sample without Ag NPs shows only weak bands that correspond to the Eu^3+^ transitions with the most distinguished ^7^F_0_→^5^L_6_ absorption placed ~ 393 nm. An enlarged inset clearly shows how Ag plasmon absorption overlaps the ^7^F_0_→^5^L_6_ absorption band of the Eu^3+^ ion [42,43].

### 3.4. Photoluminescence Measurements

**Figure 5:**
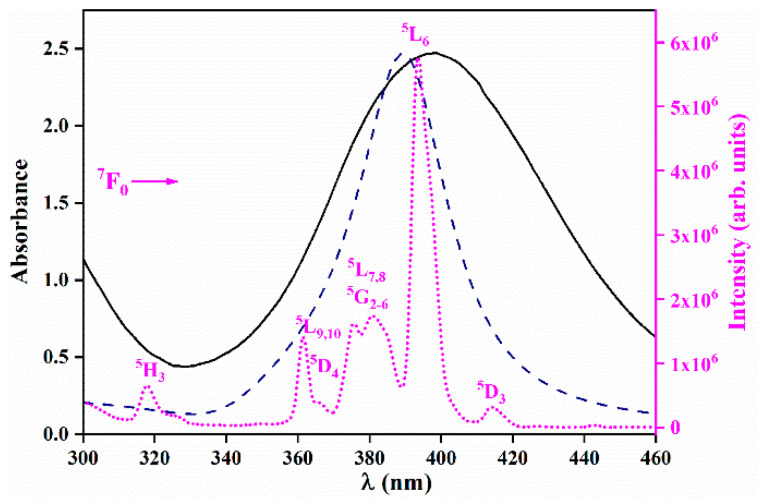
Ag NPs’ plasmonic absorption (black solid line) and the theoretical extinction efficiency curve for the 6 nm spherical Ag particles in water (blue dashed line), overlapping with the excitation spectrum of the LPO–Ag0.6 representative sample (λ_em_ = 611nm, pink dotted line).

Figure 5 presents the UV–VIS absorption spectrum of the representative Ag colloid (1 × 10^−3^ M) together with the theoretical extinction efficiency curve for 6 nm spherical Ag particles in water and the excitation spectrum of the representative LPO–Ag0.6 sample. The UV–VIS absorption spectra of all silver colloids used for the synthesis (1 × 10^−4^ M; 2 × 10^−4^ M; 1 × 10^−3^ M; 2 × 10^−3^ M; 3 × 10^−3^ M) are given in the Supporting Material as Figure S1. The excitation spectrum of the LPO–Ag0.6 sample (λ_em_ = 611nm) consists of several sharp lines that correspond to the transitions within the 4*f* ^6^ configuration of Eu^3+^ ions. The strongest Eu^3+^ line in the LPO–Ag0.6 excitation spectrum (^7^F_0_ ➝ ^5^L_6_ at 393 nm) overlaps with the silver nanoparticles’ plasmon resonance, making this combination prospective for coupling. The incident light wave resonates with oscillations of the surface electron plasma in the Ag NPs at a certain resonance frequency/wavelength. The light-induced, so-called surface plasmon, electric field spreads outside the Ag NPs and influences the neighbouring Eu^3+^ ions by increasing their light absorption. Thus, the Eu^3+^ ions will absorb more and therefore emit more light. In that way, by the incorporation of Ag NPs into the LPO host, the intensity of Eu^3+^ emission could be enhanced [23].

**Figure 6:**
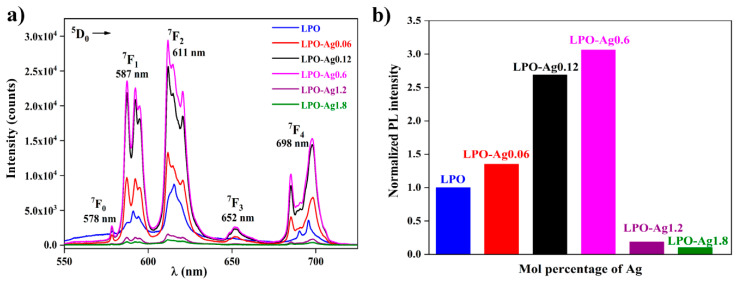
(**a**) Photoluminescence emission spectra of the LPO and Ag/La_0.95_Eu_0.05_PO_4_ nanostructures; (**b**) the normalized photoluminescent (PL) intensity as a function of the Ag NP concentration in the LPO and Ag/La_0.95_Eu_0.05_PO_4_ nanostructures showing the maximum enhancement factor ~3.

Figure 6a shows the photoluminescence spectra of all six samples, the LPO and the five samples with different concentrations of Ag NPs. Five characteristic Eu^3+^ emission bands associated with ^5^D_0_ → ^7^F_J_ (J = 0, 1, 2, 3, 4) spin forbidden *f*–*f* transitions, are visible around 578, 587, 611, 652, and 698 nm, respectively. It is well known that the ^5^D_0_ → ^7^F_1_ transition of Eu^3+^ ion is the parity-allowed magnetic dipole transition (ΔJ = 1) and its intensity does not vary with the host. On the other hand, red ^5^D_0_ → ^7^F_2_ electric dipole transition (ΔJ = 2) is highly sensitive to the local environment, and its intensity decreases with an increase in the symmetry of the crystal field around the europium ion. In the given structure, the Eu^3+^ ion replaces La^3+^ in the non-centrosymmetric *C*1 crystallographic site. It is generally acknowledged that the observation of the dominant ^5^D_0_ → ^7^F_2_ line and completely forbidden ^5^D_0_ → ^7^F_0_ transition indicates that Eu^3+^ ion is located in a structural site without a inversion centre (such as *C*1) [42,43]. This is in agreement with the observed photoluminescence spectra where the red emission arising from the ^5^D_0_ → ^7^F_2_ transition is more intense compared to the ^5^D_0_ → ^7^F_1_ emission.

One of the conditions for enhanced luminescence effect is the precise control of a distance between the plasmon particles and the emitter. Here, the distance is indirectly varied by changing the Ag nanoparticle’s concentration in the La_0.95_Eu_0.05_PO_4_ host. Figure 6b shows the normalized integrated area of the photoluminescent emission spectra as a function of Ag NPs mol % in nanostructures. The emission intensity increases up to 0.6 mol % of Ag NPs while higher Ag concentrations cause emission intensity decrease. When the concentration of Ag NPs is ≤ 0.6 mol %, phosphor particles are located in the vicinity of plasmon particles which results in the increase in emission intensity due to more effective light absorption. The optimal Ag NP concentration is 0.6 mol % and it results in the ~ 3 emission enhancement factor (distribution of the light-induced electric field near Ag nanoparticles calculated by the means of the discrete dipole approximation, given and discussed in the Supporting Material as Figure S2). On the other hand, the high Ag NP concentrations (>0.6 mol %) cause the close packing and aggregation of the plasmon particles leading to:

(i) the displacement of La_0.95_Eu_0.05_PO_4_ phosphor particles from the vicinity of the metal particles and consequently a luminescence efficiency decrease [20];
(ii) the reabsorption of emitted light, due to the electromagnetic coupling between the neighbouring particles in the aggregates. Quinten et al. reported that the Ag aggregate spectra clearly show single-particle resonance splitting into new resonances, most of which contribute at longer wavelengths (~500–600 nm) than the resonance wavelength of the single particle (~400 nm). Thus, the strongest Eu^3+^ excitation line (~393 nm) does not overlap efficiently with the silver nanoparticles’ plasmon resonance [44].

## 4. Conclusions

The brightness of phosphors can be increased due to stronger light absorption when phosphor particles are located in the vicinity of plasmon particles, for example noble metal clusters. Herein, we showed that the surface plasmon resonance facilitated the enhancement of the Eu^3+^-doped LaPO_4_ emission intensity by the addition of Ag nanoparticles. One of the conditions for the enhancement of the phosphors’ emission intensity was the precise control of the distance between the plasmon particles and the phosphor. The distance is indirectly varied by the Ag NPs concentration in the structure. When the Ag NPs concentration is low, i.e., when there is a small number of Ag NPs, the distance between the Ag and phosphor particles is long. With the Ag NPs concentration increase, the distance becomes shorter, reaching the optimum at 0.6 mol % Ag and resulting in a three times larger value than the initial photoluminescent intensity. One could expect that with the further increase in concentration (and number of NPs), the distance would be even shorter, leading to further photoluminescent intensity enhancement. However, that is not the case due to the close packing and aggregation of the plasmon particles which causes both the reabsorption of emitted light and the displacement of the La_0.95_Eu_0.05_PO_4_ phosphor particles from the vicinity of the metal particles. The distance between the phosphor particles and the Ag NPs can also be adjusted by coating phosphor particles with a different thickness SiO_2_ shell, which will be a subject of our future work.

## Supplementary Materials

The following are available online at https://www.mdpi.com/1996-1944/13/14/3071/s1, Figure S1: UV–VIS absorption spectra of the different concentrations of silver colloids, Figure S2: Distribution of the square of the electric field amplitude (E/E_0_)^2^ for 6 nm spherical Ag nanoparticles (NPs) in (a) water and in (b) La_0.95_Eu_0.05_PO_4_ (LPO).
