# Supplementary material for: Surface Plasmon Enhancement of Eu3+ Emission Intensity in LaPO4/Ag Nanoparticles
Source: Materials (Basel). 2020 Jul 10;13(14):3071. doi: 10.3390/ma13143071 (PMC7412108; doi:10.3390/ma13143071)
Supplement: Supplementary file 1 [file materials-13-03071-s001.pdf]

materials

Supplementary Materials

Surface Plasmon Enhancement of Eu^{3+} Emission Intensity in $LaPO_4$/Ag Nanoparticles

Figure S1 shows UV–VIS absorption spectra of different concentrations of silver colloids (1×10^{-4} M; 2×10^{-4} M; 1×10^{-3} M; 2×10^{-3} M; 3×10^{-3} M). The absorbance change with the concentration variation is clearly visible. However, there is no observable shift of the absorption maximum to longer wavelengths compared to the ~400 nm peak.

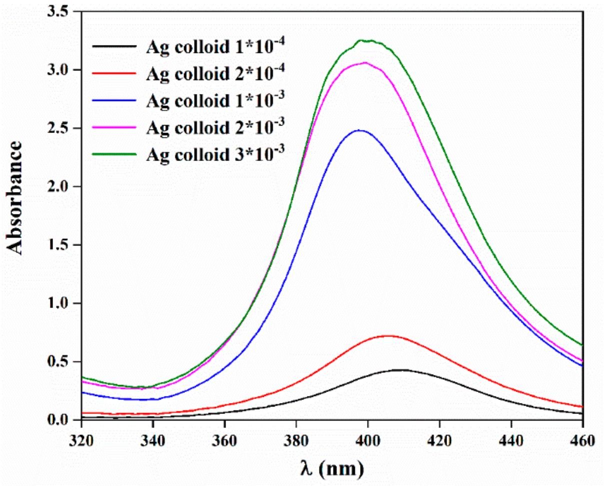

Figure S1. UV–VIS absorption spectra of the different concentrations of silver colloids.

The distribution of the light-induced electric field near the Ag nanoparticles was calculated by the means of the discrete dipole approximation. Since the fluorescence enhancement is proportional to the local field enhancement $(E/E_0)^2$, to account for all the possible silver to $La_{0.95}Eu_{0.05}PO_4$ (LPO) orientations, we calculated the near-field for 6 nm nanoparticles (NPs) fully embedded in water and LPO (Figure S2). As expected, the dipole absorption by the Ag NPs results in the enhancement of $(E/E_0)^2$ by an order of magnitude in water, and about five times in LPO. These are, of course, limiting values, and the effective enhancement is between them. On the other hand, in Figure S2b it can be seen that the enhanced field propagates by ~3 nm in the LPO, and therefore can be observed three times the enhancement of the photoluminescence in the LPO–Ag0.6 sample, due to the inhomogeneous local field in the Ag/$La_{0.95}Eu_{0.05}PO_4$ nanostructures.

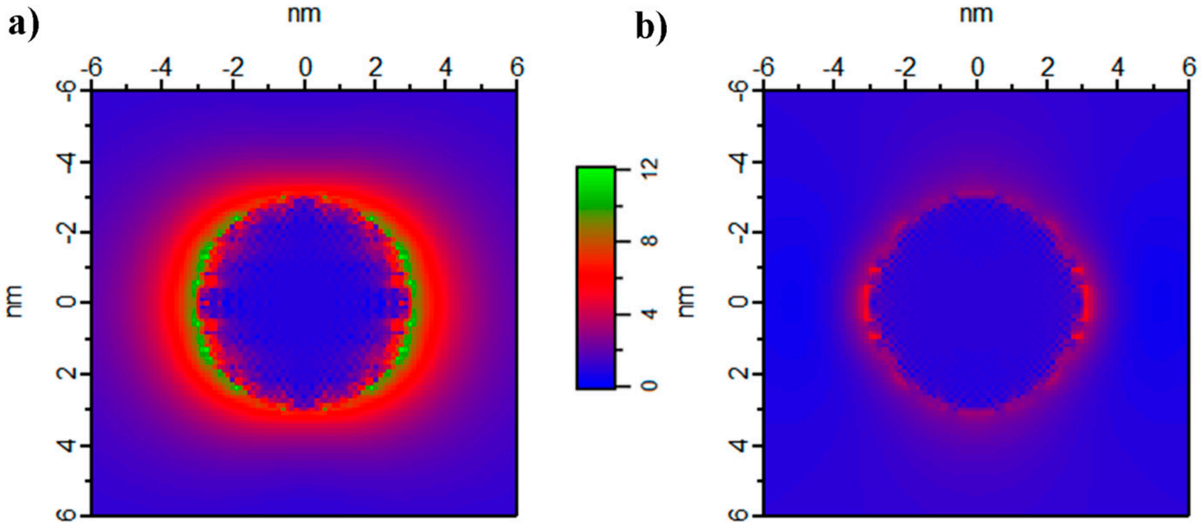

Figure S2. Distribution of the square of the electric field amplitude $(E/E_0)^2$ for 6 nm spherical Ag nanoparticles (NPs) in (**a**) water and in (**b**) $La_{0.95}Eu_{0.05}PO_4$ (LPO).

© 2020 by the author. Licensee MDPI, Basel, Switzerland. This article is an open access article distributed under the terms and conditions of the Creative Commons Attribution (CC BY) license (http://creativecommons.org/licenses/by/4.0/).
